# Intrauterine Growth Restriction Induces Adulthood Chronic Metabolic Disorder in Cardiac and Skeletal Muscles

**DOI:** 10.3389/fnut.2022.929943

**Published:** 2022-07-22

**Authors:** Ping Li, Lewei He, Yue Lan, Jie Fang, Zhenxin Fan, Yifei Li

**Affiliations:** ^1^Key Laboratory of Birth Defects and Related Diseases of Women and Children of MOE, State Key Laboratory of Oral Diseases, National Clinical Research Center for Oral Diseases, Department of Pediatrics, West China Second University Hospital, West China Hospital of Stomatology, Sichuan University, Chengdu, China; ^2^Key Laboratory of Bio-Resources and Eco-Environment, Ministry of Education, College of Life Sciences, Sichuan University, Chengdu, China

**Keywords:** IUGR, metabolic disorder, mitochondria, inflammation activity, low protein diet

## Abstract

**Objective:**

Although population-based studies of intrauterine growth restriction (IUGR) demonstrated a series of postnatal complications, several studies identified that IUGR could definitely cause dysfunction of metabolism of cardiac and skeletal muscles in the perinatal period and early life. However, it is still unknown if such metabolic alternation would remain for long term or not, and whether normal protein diet administration postnatally would protect the IUGR offsprings from a “catch-up growth” and be able to reverse the premature metabolic remodeling.

**Materials and Methods:**

We established an IUGR rat model with pregnant rats and a low-protein diet, and the developmental phenotypes had been carefully recorded. The cardiac and skeletal muscles had been collected to undergo RNA-seq.

**Results:**

According to a series of comparisons of transcriptomes among various developmental processes, programmed metabolic dysfunction and chronic inflammation activity had been identified by transcriptome sequencing in IUGR offsprings, even such rats presented a normal developmental curve or body weight after normal postnatal diet feeding.

**Conclusion:**

The data revealed that IUGR had a significant adverse impact on long-term cardiovascular function in rats, even they exhibit good nutritional status. So that, the fetal stage adverse events would encode the lifelong disease risk, which could hide in young age. This study remaindered that the research on long-term molecular changes is important, and only nutrition improvement would not totally reverse the damage of IUGR.

## Introduction

With the rapid development of the “Developmental Origins of Health and Disease” theory, associations between fetal-stage adverse events and adulthood diseases have been well established (1, 2). The placenta and womb generate an intrauterine environment that protects the fetus from harmful external stimulation, ideally ensuring normal fetal development (3). Intrauterine growth restriction (IUGR) is defined as pathological outcomes due to insufficient nutrition resulting in a body weight that is in the lowest tenth percentile of gestational age. Population-based IUGR studies have identified a series of associated postnatal complications, including disordered skeletal muscle maturation, cerebral injuries, metabolic dysfunction, elevated risk of diabetes, and cardiovascular diseases (4–15). Exposure to an adverse intrauterine environment also leads to a higher incidence of neonatal mortality and morbidity, which are reportedly associated with metabolic disorders.

Several studies indicate that IUGR can cause cardiac and skeletal muscle dysfunction of metabolism during the perinatal period and early life (16, 17). In a recent study, Maréchal et al. (18) detected an alteration in lipid metabolic gene reprogramming and changes in long-chain fatty acid profile with increased oxidative stress in cardiac muscle samples from IUGR rats at birth, which provided an essential molecular basis secondary to IUGR. Notably, however, whether such metabolic alteration remains long-term is currently unknown. It is also not known whether a normal protein diet postnatally would protect IUGR offspring from “catch-up growth” and be able to reverse the prenatal metabolic remodeling.

In the current study, an IUGR rat model was established by administering a low-protein diet prenatally to investigate whether normal protein diet supplementation postnatally would attenuate prenatal metabolic remodeling. Transcriptome sequencing was used to investigate potential myocardial and skeletal muscle disorders in IUGR rats compared to control rats. Attempts were made to identify whether programmed metabolic dysfunction and chronic inflammation occurred even in IUGR rats that were fed a normal diet postnatally and exhibited a normal developmental curve. A different transcriptional profile associated with an obese phenotype was also identified.

## Materials and Methods

### Establishment of an Intrauterine Growth Restriction Animal Model

All animal procedures were performed following protocols approved by the Institutional Animal Care and Use Committee of West China Second University Hospital, Sichuan University. Female and male Sprague-Dawley rats were fed an *ad libitum* standard laboratory chow diet (20% protein) and tap water. Female rats were mated overnight with a male after habitation, and copulation was verified the next morning by the presence of spermatozoa in vaginal smears. Pregnant rats were maintained on a 20% normal protein diet (control) or an isocaloric low-protein (LP, 8%) diet, as described (1), and the content of ingredients is listed in Supplementary Table 1. Both diets were purchased from Xietong (Jiangsu, China). Cross-fostering techniques were used to generate protein-restricted offspring during gestation or lactation. At delivery, pups born from restricted mothers were adopted randomly by restricted mothers (R) or control mothers (C) to create the experimental groups RR (*n* = 20) and RC (*n* = 20), meanwhile pups born from control mothers were suckled by control foster mothers (C) to create the control group CC (*n* = 20), in which the first letter refers to maternal dietary intake during gestation and the second letter refers to maternal dietary intake during lactation. All animals were weaned onto a standard diet containing 20% protein at 21 days of age and remained on this diet until the end of the study. Body weights and length were recorded on days 3, 7, 14, 21, and per week for 3 months, and per month from 3 to 9 months. Male pups in each group were randomly selected at the month ages of 3 and 9 after delivery and were rapidly euthanized between 9 and 11 a.m. by CO_2_ inhalation, and we ended up selecting 30 rats for the experiment. Compared with female rats, male rats are more prone to obesity, abnormal glucose tolerance, and insulin resistance (19). Glucose and lipid metabolism in female rats may be regulated by sex hormone levels (20). Heterogeneity between males and females is not the focus of this study. Therefore, female rats were not included in the study. At the postmortem, skeletal muscles and cardiac muscles were removed, snap-frozen in liquid nitrogen, and stored at −80°C until required for analysis.

### Sample Collection

We collected 28 samples from the cardiac muscle and skeletal muscle of rats, respectively. Among them, there were 13 samples from the 3-month-old rats and 15 samples from the 9-month-old rats, both from cardiac muscle and skeletal muscle.

The cardiac muscle and skeletal muscle samples were grouped according to the nutritional conditions, months of age, and weight of the rats. By feeding rats that received different nutritional restrictions and recording their body weights at 3 or 9 months, we finally divided both the cardiac muscle samples and skeletal muscle samples into nine groups (Supplementary Table 2).

### Library Preparation and RNA Sequencing

After the qualification of 56 RNA samples, we used magnetic beads with Oligo(dT) to enrich eukaryotic mRNA. Subsequently, a fragmentation buffer was added to break the mRNA into short fragments. Using mRNA as a template, one-strand cDNA was synthesized with six-base random primers (random hexamers), and then buffer, dNTPs, and DNA polymerase I and RNase H were added to synthesize two-strand cDNA. Then, AMPure XP beads were used to purify double-stranded cDNA. The purified double-strand cDNA was first repaired, and then AMPure XP beads were used for fragment size selection. Finally, PCR amplification was performed, and AMPure XP beads were used to purify the PCR products to obtain the final library. After the library was constructed, Qubit 2.0 was used for preliminary quantification, diluted the library, and then Agilent 2100 was used to detect the size of the insert in the library. After the library was qualified, the different libraries were pooled to flowcell according to the effective concentration and target data volume requirements. After the cBOT was clustered, the Illumina high throughput sequencing platform NovaSeq 6000 was used for sequencing.

### Read Alignment and Quality Control

We obtained the high-quality reads using the NGS QC Toolkit v2.3.3 (21). Processed reads from rats were mapped to the reference genome (Rnor_6.0, GCA_000001895.4) using HISAT2 v2.1.0 (22). We assembled transcriptomes and obtained raw read counts for each gene and transcript by StringTie v1.3.6 (23). Finally, we obtained the expression value of transcripts per million (TPM) for each gene using a reference annotation file. We downloaded the genome sequences and annotations from Ensembl^1^.

### Identification of Differentially Expressed Genes and Enrichment Analyses

We used the DESeq2 R (3.6.2) package to perform the differential expression analysis on the different groups of rats (24). The groups were divided according to the sampling site, age, weight, and nutritional restrictions of the rats. We chose all statistical test results for multiple testing with the Benjamini-Hochberg false discovery rate (FDR_≤_ 0.05) and determined the significant differences in gene expression with an absolute value of log2-fold change (|log2FC| ≥ 1). Furthermore, we performed Gene Ontology (GO) and Kyoto Encyclopedia of Genes and Genomes (KEGG) enrichment analyses by g:Profiler^2^ (25). The GO and KEGG enrichment analyses could determine the biological function of the Differentially expressed genes (DEGs) in rats (*Rattus norvegicus*). We plotted the bar chats of the GO enrichment analysis and bubble charts of the KEGG enrichment analysis using the ggplot2 R package (26).

### Differentially Expressed Gene Flow Gene Ontology Trees

The hierarchical relationships between GO categories in GO trees were obtained using the GO.db R package (version 3.10.0). In the cardiac muscle samples of rats, we investigated the distribution of six DEGs within the metabolic process (GO:0008152) between the RC_3_norm and CC_3_norm. Between the RC_9_norm and CC_9_norm of the cardiac muscle samples, we investigated the distribution of 18 DEGs within the inflammatory response (GO:0006954), 7 DEGs within the mitochondrion organization (GO:0007005), and 19 DEGs within the response to lipid (GO:0033993). Between the RR_9_mal and CC_9_ovob of the cardiac muscle samples, we investigated the distribution of 36 DEGs within the mitochondrion organization (GO:0007005). In the skeletal muscle samples of rats, we investigated the distribution of 10 DEGs within the metabolic process (GO:0008152) between the RC_9_norm and CC_9_norm. Between the RR_9_mal and CC_9_ovob of the skeletal muscle samples, we investigated the distribution of 13 DEGs within the mitochondrion organization (GO:0007005).

### Statistics

The data are presented as the mean ± standard deviation and individual data points. A two-way analysis of variance was used to assess the statistical significance of the differences between groups, followed by the Student–Newman–Keuls test for multiple comparisons. *P* < 0.05 was considered as a statistically significant difference. Statistical analysis was performed using the SPSS software version 21.0 (IBM Corporation, Armonk, NY, United States).

## Results

### Low-Protein Diet in Pregnancy Caused Fetal Intrauterine Growth Restriction, and Postnatal Normal Diet Could Stimulate Catch-Up Growth

**FIGURE 1:**
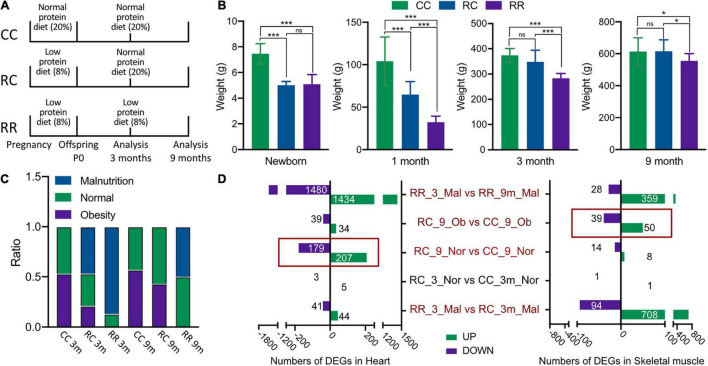
Low-protein diet in pregnancy resulted in fetal IUGR. The impacts of IUGR remain for long-term to impair the metabolism of cardiac and skeletal muscle. **(A)** The feeding protocols among different groups and the time point for sample harvest. **(B)** The average body weights among RC, RR, and CC groups at different follow-up time points, which demonstrated that the normal protein diet feeding would help the offsprings with IUGR to achieve standard adulthood body weight. **(C)** The formation of nutrition status of rats among various experimental groups. **(D)** Number of differentiated expressed genes (DEGs) identified in five comparisons of cardiac and skeletal muscles. *p < 0.05, ***p < 0.001.

Initially, 40 pregnant rats were provided a low-protein diet (8%) to establish the IUGR intervention, and control pregnant rats were provided a normal protein diet (20%). IUGR rats were randomized into two subgroups: those whose offspring were fed a normal postnatal diet for 9 months (RC group) and those whose offspring were fed a low-protein diet for 9 months (RR group). Offsprings from the control pregnant rats were fed a normal diet for 9 months postnatally, and thereafter constituted the control offspring (CC group). The follow-up period was 9 months (Figure 1A). Nutritional status and body weight development were compared among the three groups, and the outcomes were classified as malnourished, normal, or obese. The growth curve had been summarized in Supplementary Figure 1.

The mean body weights of newborns were 7.39 ± 0.94 g in the CC group, 5.01 ± 0.30 g in the RC group, and 5.09 ± 0.75 g in the RR group (Supplementary Table 2). The mean body weights of newborns in the RC and RR groups were significantly lower than that of the CC group, whereas there was no significant difference between the RC group and the RR group. At 1 month postnatally, the RC group exhibited catch-up growth, as evidenced by a larger mean body weight (64.85 ± 15.16 g) than the RR group (32.23 ± 7.26 g); but it was still lower than that of the CC group (104.10 ± 28.61 g). At 3 months, the RC group exhibited normal body weight (348.05 ± 46.08 g) that was comparable to that of the CC group (373.76 ± 26.92 g), but the RR group did not (283.33 ± 18.89 g). This indicated that normal protein diet administration postnatally could facilitate catch-up growth in IUGR offsprings within 3 months. At 9 months postnatally, there was still no significant difference between RC rats (615.03 ± 72.74 g) and CC rats (613.13 ± 87.50 g), whereas RR rats did exhibit significantly lower body weight (555.17 ± 45.48 g) (Figure 1B).

Body mass indexes were then calculated for every rat, and they were classified as malnourished (mal), normal (norm), or overweight/obese (ob). At 3 months, the CC group contained 50% normal rats and 50% obese rats (Figure 1C). The RC group contained 50% malnourished rats, 30% normal rats, and 20% obese rats (Figure 1C). Most rats in the RR group (85%) were classified as malnourished (Figure 1C). At 9 months, there was no significant difference between the ratios of different nutrition statuses in the RC and CC groups, and there were no longer any malnourished rats in the RC group, but approximately 50% of the rats in the RR group remained malnourished (Figure 1C). Thus, the developmental measurements indicated that administration of a normal protein diet postnatally facilitated catch-up growth in the IUGR offspring, such that they exhibited normal average body weight from 3 months postnatally. Notably, it was still unknown whether a risk of metabolic disorder was caused by poor nutrition or was already encoded as a program that would remain in offspring even after their malnourished condition had improved. Thus, transcriptome analysis was conducted under the same nutritional conditions in rats with and without IUGR.

### Differentially Expressed Genes in Same Phenotype Offspring Among Different Diet Supplementations

Twenty-eight cardiac muscle samples and 28 skeletal muscle samples were collected from 28 rats. Thirteen samples of each type were collected at the age of 3 months and 15 samples of each type were collected at the age of 9 months. A total of 1,478,261,292 bp paired-end clean reads were obtained from the 56 samples. All clean reads were aligned to the reference genome (Rnor_6.0) with an average mapping rate of 96.18%. After removing low-expression genes, these reads were assembled into 20,612 known rat (*Rattus norvegicus*) genes. All sample data were then collated based on the rat group allocations (the nutritional conditions they were experimentally subjected to) and their body weight data at the ages of 3 and 9 months.

To assess the effects of IUGR on the long-term development of cardiac muscle and skeletal muscle in rats, the samples were named according to the experimental conditions they were derived from, with or without IUGR, and five comparisons were made among nine groups. The nine groups are listed in Supplementary Table 2, and the five comparisons are as follows: (1) RR_3_mal vs. RC_3_mal; (2) RC_3_norm vs. CC_3_norm; (3) RR_3_mal vs. RR_9_mal; (4) RC_9_norm vs. CC_9_norm; and (5) RC_9_ovob vs. CC_9_ovob. Differentially expressed genes identified by the same bioinformatics pipeline in cardiac muscle and skeletal muscle are shown in Figure 1D. DEGs with higher expression levels in one group compared to another were deemed “upregulated,” and conversely those with lower expression levels were deemed “downregulated.” At 3 months of age, malnourished offspring fed a low-protein diet exhibited significant gene expression changes in skeletal muscle (RC_3_mal vs. RR_3_mal, 708 upregulated genes, and 94 downregulated genes). Changes were also evident in cardiac muscle, but less number of significantly differentiated genes had been recorded in the heart compared to skeletal muscle (RC_3_mal vs. RR_3_mal, 41 upregulated and 44 downregulated). Comparisons between malnourished rats at 3 months and those at 9 months revealed significant differences (RR_3_mal vs. RR_9_mal). Interestingly, at 3 months of age, normal nutritional status rats exhibited similar transcriptional profiles in comparisons between RC and CC groups. Despite this, dramatic gene expression changes were evident in cardiac muscle samples of rats with normal body weight (RC_9_norm vs. CC_9_norm, 207 upregulated genes, and 179 downregulated genes) and skeletal muscle samples of obese rats (RC_9_ovob vs. CC_9_ovob, 50 upregulated genes, and 39 downregulated genes) at the age of 9 months among normal nutrition rats. Thus, although a normal protein diet administered postnatally facilitated normal weight gain and body development in IUGR offspring, there was no significant influence in the short term (3 months). The encoding effects of IUGR appeared in the long term (9 months), even in rats that exhibited normal adulthood body development and weight gain.

### Intrauterine Growth Restriction Activates Chronic Inflammation in Cardiac Muscle

**FIGURE 2:**
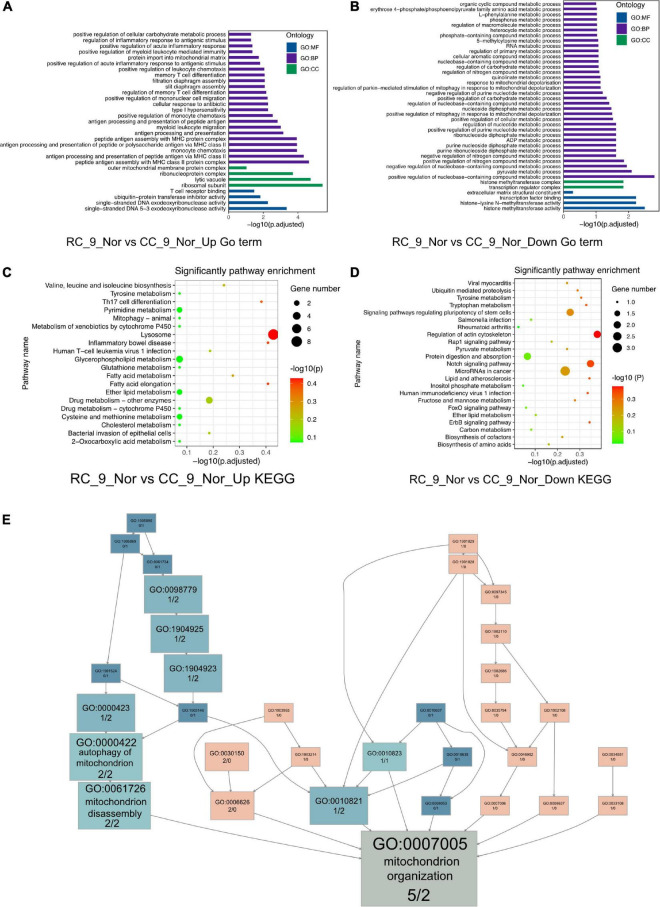
The GO and KEGG analysis of the DEGs (RC_9_Nor vs. CC_9_Nor) in the cardiac muscle samples of the rats. **(A)** The GO enrichment analysis of upregulated DEGs in RC_9_Nor and downregulated DEGs in CC_9_Nor. **(B)** The GO enrichment analysis of downregulated DEGs in RC_9_Nor and downregulated DEGs in CC_9_Nor. **(C)** The KEGG enrichment analysis of upregulated DEGs in RC_9_Nor and downregulated DEGs in CC_9_Nor. **(D)** The KEGG enrichment analysis of downregulated DEGs in RC_9_Nor and downregulated DEGs in CC_9_Nor. **(E)** The GO tree of 7 mitochondrion-related DEGs in mitochondrion organization (GO:0007005).

**FIGURE 3:**
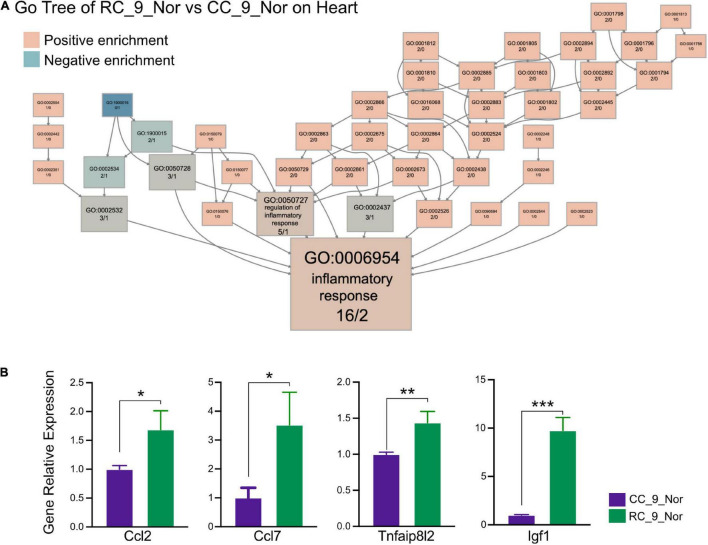
IUGR activates chronic inflammation in the heart. **(A)** The GO tree of RC_9_Nor vs. CC_9_Nor on the cardiac muscle. The box numbers represented the relative amount of DEG in the category. The box color equated to the numbers of upregulated and downregulated DEGs, with upregulated represented by pink and downregulated equaling blue, which indicated chronic inflammation activity existing in the heart due to IUGR. **(B)** The verification of important genes in GO:0006954 between these two groups by qPCR. *p < 0.05, **p < 0.01, ***p < 0.001.

Significantly different gene expression profiles were identified in normal body weight rats with and without IUGR. To investigate what had been altered due to IUGR, GO, and KEGG enrichment was performed in cardiac muscle of RC_9_norm samples and CC_9_norm samples. In GO enrichment, 207 upregulated DEGs in RC_9_norm samples were involved in peptide antigen assembly with MHC class II protein complex (GO:0002503), regulation of inflammatory responses to antigenic stimuli (GO:0002861), and positive regulation of cellular carbohydrate metabolism (GO:0010676) (Figure 2A and Supplementary Table 3). Conversely, 179 downregulated DEGs were involved in positive regulation of nucleobase-containing compound metabolism (GO:0045935), positive regulation of mitophagy in response to mitochondrial depolarization (GO:0098779), and 5-methylcytosine metabolism (GO:0019857) (Figure 2B and Supplementary Table 4). In KEGG enrichment, the upregulated DEGs in RC_9_norm samples were involved in lysosomes (KEGG:04142), inflammatory bowel disease (KEGG:05321), and glycerophospholipid metabolism (KEGG:00564) (Figure 2C and Supplementary Table 3). Downregulated DEGs in RC_9_norm samples were involved in the regulation of the actin cytoskeleton (KEGG:04810), tryptophan metabolism (KEGG:00380), and fructose and mannose metabolism (KEGG:00051) (Figure 2D and Supplementary Table 4). Interestingly, GO tree analysis revealed that inflammatory responses were enriched in RC cardiac muscle samples at 9 months (Figures 3A,B), whereas mitochondrial organization was disrupted in the long term (Figure 2E).

### Continuous Low-Protein Diet Aggravates Mitochondrial Dysfunction in Cardiac Muscle

**FIGURE 4:**
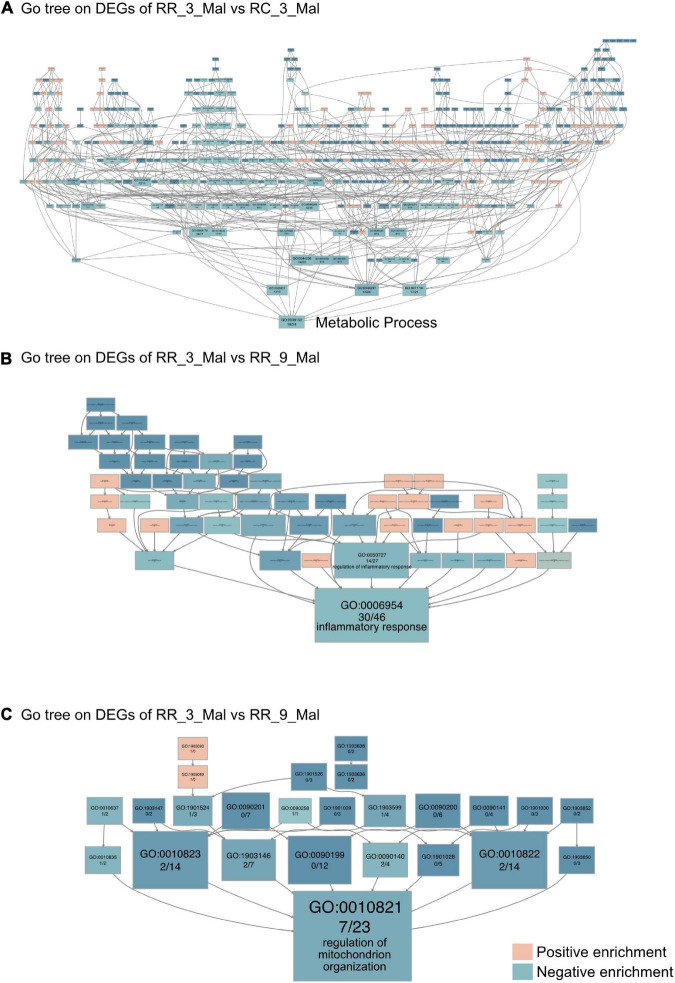
Continuous low-protein diet aggravates mitochondrial dysfunction in the heart. **(A)** The GO tree of RR_3_Mal vs. RC_3_Mal on the heart. **(B)** The GO tree of 76 inflammatory-related DEGs in inflammatory response (GO:0006954). **(C)** The GO tree of 30 mitochondrion-related DEGs in the regulation of mitochondrion organization (GO:0010821). The box color equated to the numbers of upregulated and downregulated DEGs, with upregulated represented by pink and downregulated equaling blue.

Gene Ontology category and the Kyoto Encyclopedia of Genes and Genomes pathway enrichment analyses were then performed based on the upregulated and downregulated DEGs identified in cardiac muscle samples from different groups to gain insights into the biological roles of these DEGs. The 44 DEGs that were upregulated in RR_3_mal samples and downregulated in RC_3_mal samples were mainly involved in metabolism-related processes such as pentose metabolism (GO:0019321), negative regulation of nucleobase-containing compound metabolism (GO:0045934), and carbohydrate metabolism (GO:0005975) (Supplementary Figure 2A and Supplementary Table 5). The 41 DEGs that were downregulated in RR_3_mal samples and upregulated in RC_3_mal samples were mainly involved in mitochondrial ATP synthesis-coupled electron transport (GO:0042775), lipid metabolism (GO:0006629), and xanthophyll metabolism (GO:0016122) (Supplementary Figure 2B and Supplementary Table 6). No convincing KEGG enrichment results were obtained. In GO tree analysis, DEGs involved in metabolic processes (GO:0008152) in RR_3_mal and RC_3_mal cardiac muscle samples indicated that a low-protein diet postnatally could cause more severe downregulation of metabolism (Figure 4A).

GO and KEGG enrichment analyses between RR_3_mal samples and RR_9_mal samples were then performed. In GO analysis, 1,434 upregulated DEGs in RR_3_mal cardiac muscle samples included genes involved in positive regulation of nucleobase-containing compound metabolism (GO:0045935), extracellular matrix organization (GO:0030198), and 5-methylcytosine metabolism (GO:0019857) (Supplementary Figure 3A and Supplementary Table 7). The 1,480 downregulated DEGs identified in RR_3_mal samples included genes involved in mitochondrial processes, such as inner mitochondrial membrane organization (GO:0007007), establishment of protein localization to the mitochondrial membrane (GO:0090151), and purine ribonucleoside triphosphate metabolism (GO:0009205) (Supplementary Figure 3B and Supplementary Table 8). In KEGG enrichment analysis, the upregulated DEGs in RR_3_mal samples included genes involved in the thyroid hormone signaling pathway (KEGG:04919), linoleic acid metabolism (KEGG:00591), and aldosterone synthesis and secretion (KEGG:04925) (Supplementary Figure 3C and Supplementary Table 7). The downregulated DEGs in RR_3_mal samples included genes involved in carbon metabolism (KEGG:01200), pyruvate metabolism (KEGG:00620), and propanoate metabolism (KEGG:00640) (Supplementary Figure 3D and Supplementary Table 8). GO tree analysis indicated negative enrichment of inflammatory responses and regulation of mitochondrial organization (Figures 4B,C) in RR_3_mal cardiac muscle samples, suggesting that chronic inflammation and metabolic disorder would occur when a low-protein diet was continuously provided.

### Intrauterine Growth Restriction Impairs Mitochondrial Function in Skeletal Muscle

**FIGURE 5:**
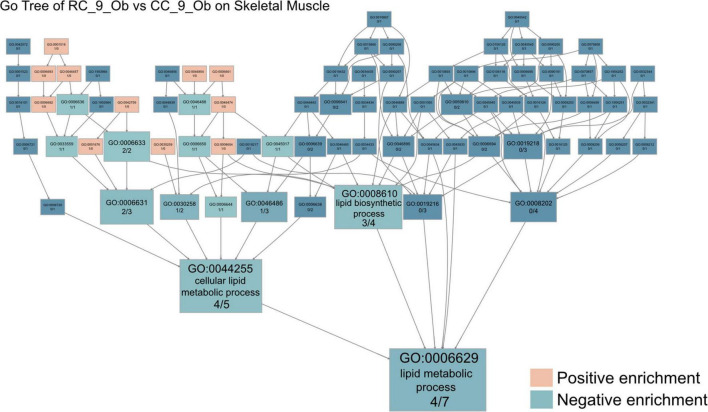
IUGR impairs mitochondrial function in skeletal muscle. The GO tree of RC_9_Ob vs. CC_9_Ob on skeletal muscle. The box numbers represented the relative amount of DEG in the category. The box color equated to the numbers of upregulated and downregulated DEGs, with upregulated represented by pink and downregulated equaling blue, revealing impaired mitochondrial function in skeletal muscles.

Among all comparisons of skeletal muscle samples, the transcriptome profile differences between obese rats in the RC and CC groups were arguably the most striking. In GO analysis, the 50 upregulated DEGs in RC_9_ovob were involved in cellular oligosaccharide metabolism (GO:0051691), quinolinate metabolism (GO:0046874), and epoxide metabolism (GO:0097176) (Supplementary Figure 4A and Supplementary Table 9). The 39 downregulated DEGs were involved in steroid metabolism (GO:0008202), lipid metabolism (GO:0006629), and inosine monophosphate metabolism (GO:0046040) (Supplementary Figure 4B and Supplementary Table 10). In KEGG analysis, the upregulated DEGs in RC_9_ovob were involved in the metabolism of xenobiotics by cytochrome P450 (KEGG:00980), nicotinate and nicotinamide metabolism (KEGG:00760) and arachidonic acid metabolism (KEGG:00590) (Supplementary Figure 4C and Supplementary Table 9). The downregulated DEGs in RC_9_ovob were involved in butanoate metabolism (KEGG:00650), purine metabolism (KEGG:00230), and retinol metabolism (KEGG:00830) (Supplementary Figure 4D and Supplementary Table 10). Both upregulated and downregulated DEGs were involved in metabolic alteration. In GO analysis performed to investigate metabolic changes in obese rats with or without IUGR, “downregulated metabolic process” was the key GO term in RC rats. Lipid metabolic processes were the dominant metabolic pathways involved in long-term skeletal muscle dysfunction due to IUGR based on Go tree analysis (Figure 5).

### Continuous Low-Protein Diet Leads to Long-Term Skeletal Muscle Dysfunction

**FIGURE 6:**
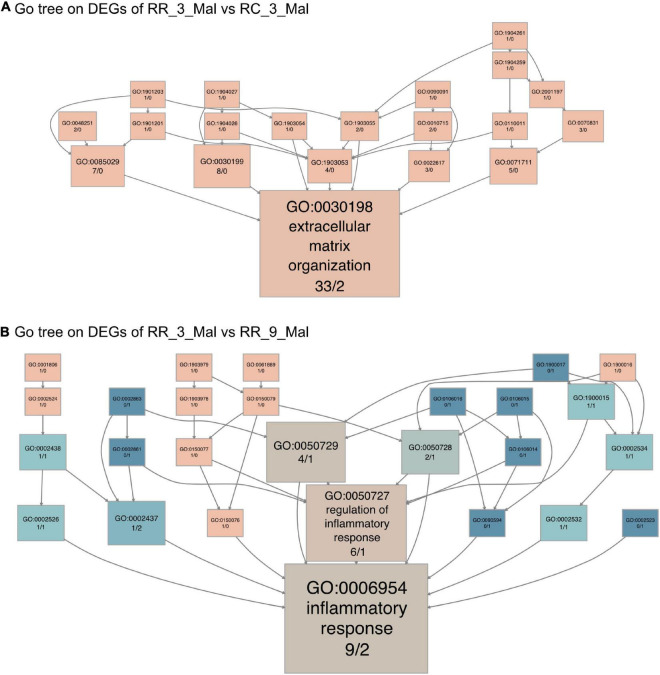
Continuous low-protein diet leads to long-term skeletal muscle dysfunction. **(A)** The GO tree of 35 extracellular matrix-related DEGs in an extracellular matrix organization (GO:0030198). **(B)** The GO tree of 11 inflammatory-related DEGs in inflammatory response (GO:0006954). The box color equated to the numbers of upregulated and downregulated DEGs, with upregulated represented by pink and downregulated equaling blue.

Similar transcriptome analyses were performed using skeletal muscle samples in various subgroup comparisons. In comparisons between RR_3_mal and RC_3_mal, 708 DEGs that were upregulated in RR_3_mal and downregulated in RC_3_mal were mainly involved in an extracellular matrix organization (GO:0030198), lipid metabolism (GO:0006629), and steroid metabolism (GO:0008202) (Supplementary Figure 5A and Supplementary Table 11). Conversely, 96 DEGs that were downregulated in RR_3_mal and upregulated in RC_3_mal were mainly involved in the regulation of glutamine family amino acid metabolism (GO:0000820) polyphosphate metabolism (GO:0006797), and mitochondria-associated endoplasmic reticulum membrane (GO:0044233) (Supplementary Figure 5B and Supplementary Table 12). In KEGG analysis, the upregulated DEGs in RR_3_mal were involved in phenylalanine metabolism (KEGG:00360), tyrosine metabolism (KEGG:00350), and the renin–angiotensin system (KEGG:04614) (Supplementary Figure 5C and Supplementary Table 11), whereas the downregulated DEGs were involved in fatty acid metabolism (KEGG:01212), cholesterol metabolism (KEGG:04979), and histidine metabolism (KEGG:00340) (Supplementary Figure 5D and Supplementary Table 12). In GO tree analysis, extracellular matrix organization was enriched in RR skeletal muscle samples at 3 months compared to RC skeletal muscle samples at 3 months (Figure 6A).

The 359 upregulated DEGs in RR_3_mal were involved in lipid metabolism (GO:0006629), steroid biosynthesis (GO:0006694), and extrinsic components of the mitochondrial outer membrane (GO:0031315) compared to RR_9_mal samples (Supplementary Figure 6A and Supplementary Table 13). The 28 downregulated DEGs in RR_3_mal were involved in inflammatory responses to antigenic stimuli (GO:0002437), thyroid hormone metabolism (GO:0042403), and collagen-containing extracellular matrix (GO:0062023) (Supplementary Figure 6B and Supplementary Table 14). In KEGG analysis, the upregulated DEGs in RR_3_mal were involved in steroid biosynthesis (KEGG:00100), glycine, serine, and threonine metabolism (KEGG:00260) and beta-alanine metabolism (KEGG:00410) (Supplementary Figure 6C and Supplementary Table 13). The downregulated DEGs in RR_3_mal were involved in the FoxO signaling pathway (KEGG:04068) and purine metabolism (KEGG:00230) (Supplementary Figure 6D and Supplementary Table 14). In GO tree analysis, inflammatory responses were enriched in RR skeletal muscle samples at 3 months compared to RR skeletal muscle samples at 9 months (Figure 6B).

## Discussion

Intrauterine growth restriction has strong effects on fetal intrauterine growth and postnatal development. It results in perinatal cardiomyocyte injuries, inflammation in microglial cells, and metabolic alteration of skeletal muscle (27). Notably, to date, most studies have focused on the short-term perinatal damage due to IUGR (18, 27). In clinical research, IUGR has been linked with a long-term risk of cardiovascular complications (17). Based on the Developmental Origins of Health and Disease theory, individuals who endure adverse events in fetal life, including malnutrition *via* a low-protein maternal diet, would be more likely to develop lifestyle-related diseases. There is thus an urgent need to evaluate the long-term effects of IUGR.

The current study investigated the effects of a low-protein diet during pregnancy concerning IUGR induction and long-term cardiac muscle and skeletal muscle functional hemostasis formation (28). Most IUGR offspring exhibited “catch-up growth” and achieved normal adulthood nutritional condition and body weight. Thus, whether improved nutritional status can reverse injuries caused by malnutrition is still a subject of debate. To answer the above question, we developed a low-protein diet rat model of IUGR. Malnutrition was identified among offspring, and metabolic dysfunction was evident at 3 months postnatally. Normal protein administration could enable the malnourished offspring to reach a normal body weight in the long term. Transcriptome analysis identified spontaneous chronic inflammatory activity and metabolic disorder in offspring with normal adult body weights despite IUGR exposure, which was not detectable in the short term.

Transcriptome sequencing and comparative analysis of cardiac muscle samples from rats undergoing different dietary conditions showed that early nutritional restriction may affect cardiac muscle metabolism, response to inflammation, and mitochondria function in rats. DOHaD supposes an inverse relationship between birth weight and long-term metabolic health, as adverse uterine events may permanently affect the metabolic function of the organs (2). Fetal growth and development consist of complex cellular processes that are highly sensitive to extracellular and intracellular stresses (2). Therefore, metabolic disease in IUGR rats can be partly attributed to the cellular stress response. A total of 386 DEGs were detected between RC_9_Nor and CC_9_Nor by differential expression analysis (207 upregulated genes in RC_9_Nor and 179 upregulated genes in CC_9_Nor). The upregulated genes in RC_9_Nor were enriched in GO terms related to the inflammatory response. Emiri Tarbit et al. found elevated levels of serotonin in patients with heart failure and that serotonin plays a role in the functioning of the pathological heart (29). Serotonin may induce valvular heart disease by stimulating valvular cells through interaction with serotonin 2B receptors (30). Serotonin production involved in inflammatory response (GO:0002351) and serotonin secretion involved in inflammatory response (GO:0002442) were significantly enriched in the upregulated genes in the RC_9_Nor group, and the gene enriched in the two GO terms was *Fcer1g*. *Fcer1g* is orthologous to human *FCER1G* and predicted to be active on the external side of plasma membrane (31). Although some IUGR rats were given a normal protein diet after birth to achieve normal body weight and nutritional status, the possibility of valvular heart disease in these rats was increased due to the upregulated expression of *Fcer1g*, leading to increased production and secretion of serotonin. The genes enriched in these three GO terms were *Fcer1g* and *Fcgr2a*. Human orthologs of *Fcgr2a* are implicated in several diseases, including autoimmune disease, dengue disease, hematologic cancer, leukopenia, and malaria. *Fcgr2a* is orthologous to human *FCGR2A* (Fc fragment of IgG receptor IIa) (32). The high expression of *Fcer1g* and *Fcgr2a* in cardiac muscle samples of RC_9_Nor rats may enhance the allergic reaction of rats and cause damage to the cardiac muscle of rats. These results suggest that IUGR rats are at increased risk of heart-related diseases and inflammation, even though they can grow to normal-weight status after birth on a normal protein diet.

Oxidative stress plays a key role in heart remodeling and the development of heart failure (33). Xanthophyll is the main non-vitamin A carotenoid with a strong antioxidant capacity, which can inhibit the activity of oxygen free radicals and prevent the damage of oxygen free radicals to normal cells. It has been proved to profoundly impact oxidative stress (34, 35). The gene enriched in the xanthophyll metabolic process (GO:0016122) and carotenoid metabolic process (GO:0016116) is *Bco2*, which is homologous to human *BCO2* (β-carotene oxygenase 2) and is expected to act in the upstream or interior of xanthophyll metabolic process, possibly active in mitochondria (36, 37). The low expression of the *BCO2* gene in the RR_3_Mal group may decrease the antioxidant capacity of the rat cardiac muscle. Therefore, continued low-protein feeding could increase the risk of heart failure in rats. Linoleic acid is an essential fatty acid that reduces blood cholesterol and prevents atherosclerosis. If linoleic acid is deficient, cholesterol will combine with some saturated fatty acids, causing metabolic disorders, deposition in the blood vessel, and gradual formation of atherosclerosis, thus causing cardiovascular and cerebrovascular diseases (38). The DEG enriched in the linoleic acid metabolic process (GO:0043651) is *Cyp4a2*. Orthologous to human *CYP4A11* (cytochrome P450 family four subfamilies A member 11) and *CYP4A22* (cytochrome P450 family four subfamilies A member 22). *Cyp4a2* plays a role in organelles in the inner membrane of cells and is involved in several biological processes, including fatty acid metabolism (39). As Cyp4a2 expression level in cardiac muscle samples of the RR_3_Mal group is lower than the RC_3_Mal group, it can be assumed that a continuous low-protein diet will inhibit the fatty acid metabolic process in cardiac muscle and increase the probability of cardiovascular and cerebrovascular diseases in rats.

Skeletal muscle growth could be limited by decreased fetal nutrient supply late in pregnancy. Reduced skeletal muscle growth was characteristic in IUGR fetuses compared with normal-weight newborns (40). Transcriptome sequencing and comparative analysis of skeletal muscle samples from rats subjected to different dietary conditions showed that early nutritional restriction might affect skeletal muscle metabolism, response to inflammation, and function of extracellular matrix tissue in rats. A total of 89 DEGs (50 upregulated in RC_9_Ob group and 39 upregulated in CC_9_Ob group; Figure 1D) were identified in skeletal muscle samples of the two groups of obese rats. Maintaining the quality and integrity of skeletal muscle is essential for the proper functioning of the musculoskeletal system and efficient nutrient absorption and storage (41). Especially in obesity, the loss of skeletal muscle mass severely weakens the musculoskeletal system and impedes movement, resulting in impaired homeostasis of glucose and lipid (41, 42). Today, there is growing evidence that fatty acids and their derived lipid intermediates play an important role in regulating skeletal muscle quality and function (43). Triglycerides are stored in lipid droplets of skeletal muscle and are hydrolyzed to produce fatty acids, which contribute significantly to energy production through β-oxidation and oxidative phosphorylation (44). Positive regulation of triglyceride biosynthetic process (GO:0010867) was significantly enriched in upregulated genes in skeletal muscle samples of rats in the CC_9_Ob group, and the enriched gene was *Srebf1*. *Srebf1* is involved in several processes, including response to peptide hormones, response to progesterone, and response to retinoic acid, and is orthologous to human *SREBF1* (45). The expression of Srebf1 was downregulated in skeletal muscle samples of rats in the RC_9_Ob group, which was not beneficial to the biosynthesis of triglyceride in rats, thus adversely affecting the energy metabolism of rats and was not conducive to the normal function of skeletal muscle. Therefore, we speculated that although IUGR rats fed with normal protein diet after birth can grow to normal or even obese weight, their skeletal muscle may still have dysfunction compared with normal rats.

A total of 802 DEGs (708 upregulated in the RR_3_Mal group and 94 downregulated in RC_3_Mal group) were identified between skeletal muscle samples of the RR_3_Mal group and RC_3_Mal group, which was the largest among the five groups of skeletal muscle samples. The upregulated genes in the RR_3_Mal were abundant in the related GO terms of an extracellular matrix organization. The skeletal muscle extracellular matrix plays an important role in the transmission, maintenance, and repair of muscle fiber force (46). The extracellular matrix is composed of a variety of substances, of which collagen fibrils are ubiquitous (47). Negative regulation of collagen fibril organization (GO:1904027) enriched gene was *Chadl*. The gene is orthologous to human *CHADL* (chondroadherin-like) and is expected to be involved in the negative regulation of chondrocyte differentiation and collagen fibrillary tissue (48). The expression of *Chadl* is upregulated in RR_3_Mal skeletal muscle samples, resulting in limited development of collagen fibril organization in RR_3_Mal rats. Therefore, we speculate that that if IUGR rats are still fed with low-protein diet after birth, the development of skeletal muscle extracellular matrix will be restricted, which will adversely affect the growth and development of skeletal muscle.

However, there were still some limitations in the current research. The first one is that the research established a very artificial IUGR model, and could also induce IUGR beyond a low-protein diet. So, the other causes that induced long-term effects on hearts and skeletal muscles require further investigation. The second one is the study only provided essential evidence on the basic presentation of offsprings post-IUGR, and the molecular mechanisms and functional validation are still required to fully illustrate the long-term injuries on hearts and skeletal muscles post-IUGR.

In summary, in the present study, IUGR was significantly associated with long-term adulthood cardiovascular complications, even in rats that exhibited good nutritional condition. This suggests that fetal-stage adverse events may encode life-long disease risks which may be hidden at a young age. This study indicates that research investigating long-term molecular changes is important and that solely improving nutrition postnatally would not totally reverse the damaging effects of IUGR. Further studies on the molecular mechanisms are required to develop more efficient methods to protect such offspring.

## Data Availability Statement

The datasets presented in this study can be found in online repositories. The names of the repository/repositories and accession number(s) can be found below: https://db.cngb.org/ and CNP0002644.

## Ethics Statement

The animal study was reviewed and approved by the West China Second University Hospital, Sichuan University.

## Author Contributions

PL and YiL conceptualized the study. PL, JF, and YiL designed the experiments. PL and JF performed the sample collection and experiments. JF, LH, YuL, and ZF analyzed the RNA-seq data. LH, YiL, JF, and ZF wrote or edited the manuscript. All authors contributed to the article and approved the submitted version.

## Conflict of Interest

The authors declare that the research was conducted in the absence of any commercial or financial relationships that could be construed as a potential conflict of interest.

## Publisher’s Note

All claims expressed in this article are solely those of the authors and do not necessarily represent those of their affiliated organizations, or those of the publisher, the editors and the reviewers. Any product that may be evaluated in this article, or claim that may be made by its manufacturer, is not guaranteed or endorsed by the publisher.

## Footnotes

1
https://asia.ensembl.org/

2
https://biit.cs.ut.ee/gprofiler/gost

## Supplementary Material

Supplementary Figure 1 The growth curve of different treated groups.

Supplementary Figure 2 The GO enrichment analysis of the DEGs (RR_3_Mal vs. RC_3_Mal) in the cardiac muscle samples of the rats. **(A)** The GO enrichment analysis of upregulated DEGs in RR_3_Mal and downregulated in RC_3_Mal. **(B)** The GO enrichment analysis of downregulated DEGs in RR_3_Mal and upregulated in RC_3_Mal.

Supplementary Figure 3 The GO and KEGG enrichment analysis of the DEGs (RR_3_Mal vs. RR_9_Mal) in the cardiac muscle samples of the rats. **(A)** The GO enrichment analysis of upregulated DEGs in RR_3_Mal and downregulated DEGs in RR_9_Mal. **(B)** The GO enrichment analysis of downregulated DEGs in RR_3_Mal and upregulated DEGs in RR_9_Mal. **(C)** The KEGG enrichment analysis of upregulated DEGs in RR_3_Mal and downregulated DEGs in RR_9_Mal. **(D)** The KEGG enrichment analysis of downregulated DEGs in RR_3_Mal and upregulated DEGs in RR_9_Mal.

Supplementary Figure 4 The GO and KEGG analysis of the DEGs (RC_9_Ob vs. CC_9_Ob) in the skeletal muscle samples of the rats. **(A)** The GO enrichment analysis of upregulated DEGs in RC_9_Ob and downregulated DEGs in CC_9_Ob. **(B)** The GO enrichment analysis of downregulated DEGs in RC_9_Ob and upregulated in CC_9_Ob. **(C)** The KEGG enrichment analysis of upregulated DEGs in RC_9_Ob and downregulated DEGs in CC_9_Ob. **(D)** The KEGG enrichment analysis of downregulated DEGs in RC_9_Ob and upregulated in CC_9_Ob.

Supplementary Figure 5 The GO and KEGG analysis of the DEGs (RR_3_Mal vs. RC_3_Mal) in the skeletal muscle samples of the rats. **(A)** The GO enrichment analysis of upregulated DEGs in RR_3_Mal and downregulated DEGs in RC_3_Mal. **(B)** The GO enrichment analysis of downregulated DEGs in RR_3_Mal and upregulated DEGs in RC_3_Mal. **(C)** The KEGG enrichment analysis of upregulated DEGs in RR_3_Mal and downregulated DEGs in RC_3_Mal. **(D)** The KEGG enrichment analysis of downregulated DEGs in RR_3_Mal and upregulated DEGs in RC_3_Mal.

Supplementary Figure 6 The GO and KEGG analysis of the DEGs (RR_3_Mal vs. RR_9_Mal) in the skeletal muscle samples of the rats. **(A)** The GO enrichment analysis of upregulated DEGs in RR_3_Mal and downregulated DEGs in RR_9_Mal. **(B)** The GO enrichment analysis of downregulated DEGs in RR_3_Mal and upregulated DEGs in RR_9_Mal. **(C)** The KEGG enrichment analysis of upregulated DEGs in RR_3_Mal and downregulated DEGs in RR_9_Mal. **(D)** The KEGG enrichment analysis of downregulated DEGs in RR_3_Mal and upregulated DEGs in RR_9_Mal. **(E)** The GO tree of 46 metabolism-related DEGs in the metabolic process (GO:0008152).
