# Supplementary material for: Intrauterine Growth Restriction Induces Adulthood Chronic Metabolic Disorder in Cardiac and Skeletal Muscles
Source: Front Nutr. 2022 Jul 22;9:929943. doi: 10.3389/fnut.2022.929943 (PMC9354130; doi:10.3389/fnut.2022.929943)
Supplement: Supplementary Figure 1 — The growth curve of different treated groups. [file Data_Sheet_1.zip › supplementary material/S_Fig_5.pdf]

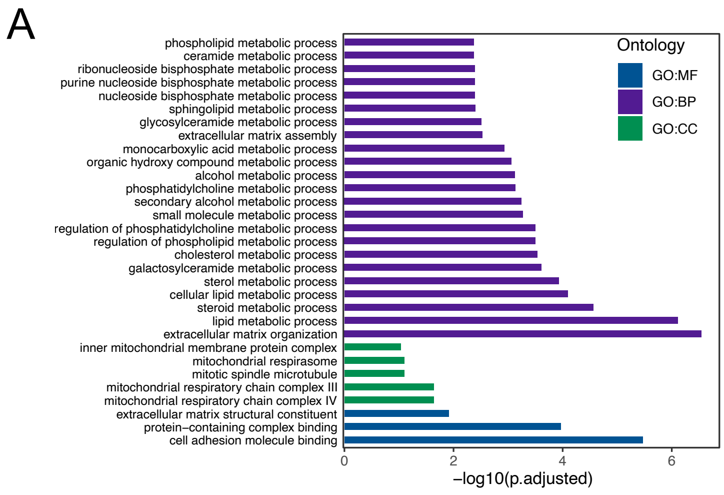

RR_3_Mal vs RC_3_Mal_Up Go term

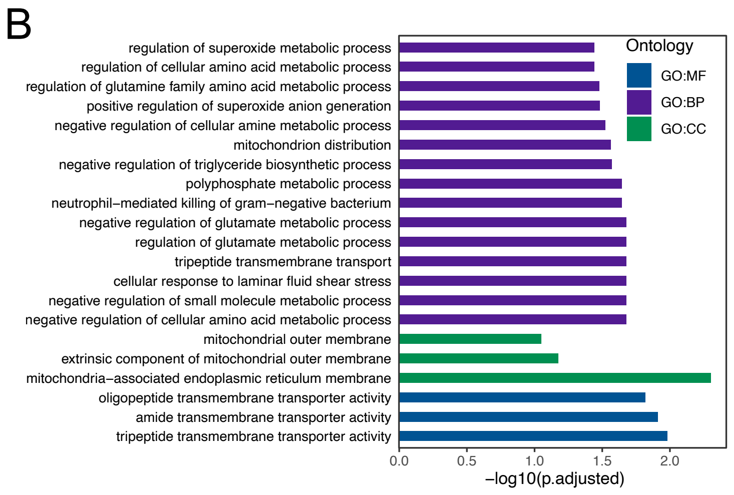

RR_3_Mal vs RC_3_Mal_Down Go term

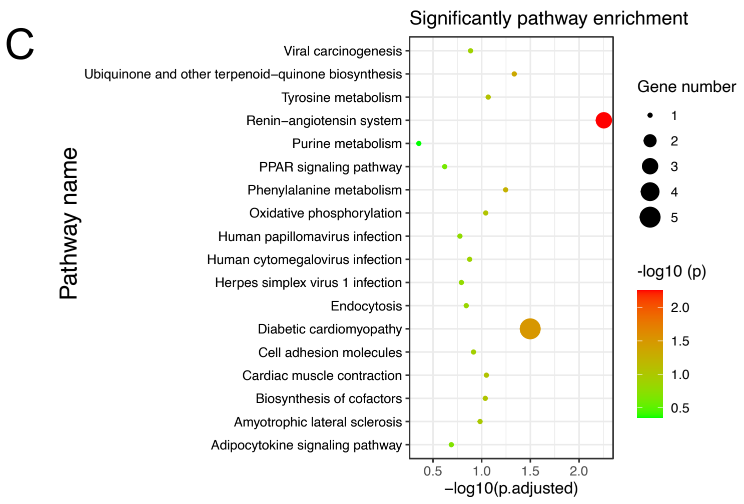

RR_3_Mal vs RC_3_Mal_Up KEGG

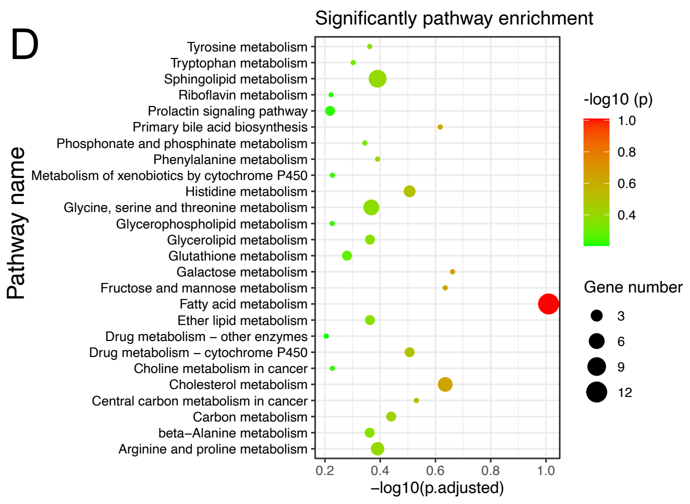

RR_3_Mal vs RC_3_Mal_Down KEGG
