# Supplementary material for: Intrauterine Growth Restriction Induces Adulthood Chronic Metabolic Disorder in Cardiac and Skeletal Muscles
Source: Front Nutr. 2022 Jul 22;9:929943. doi: 10.3389/fnut.2022.929943 (PMC9354130; doi:10.3389/fnut.2022.929943)
Supplement: Supplementary Figure 1 — The growth curve of different treated groups. [file Data_Sheet_1.zip › supplementary material/S_Fig_3.pdf]

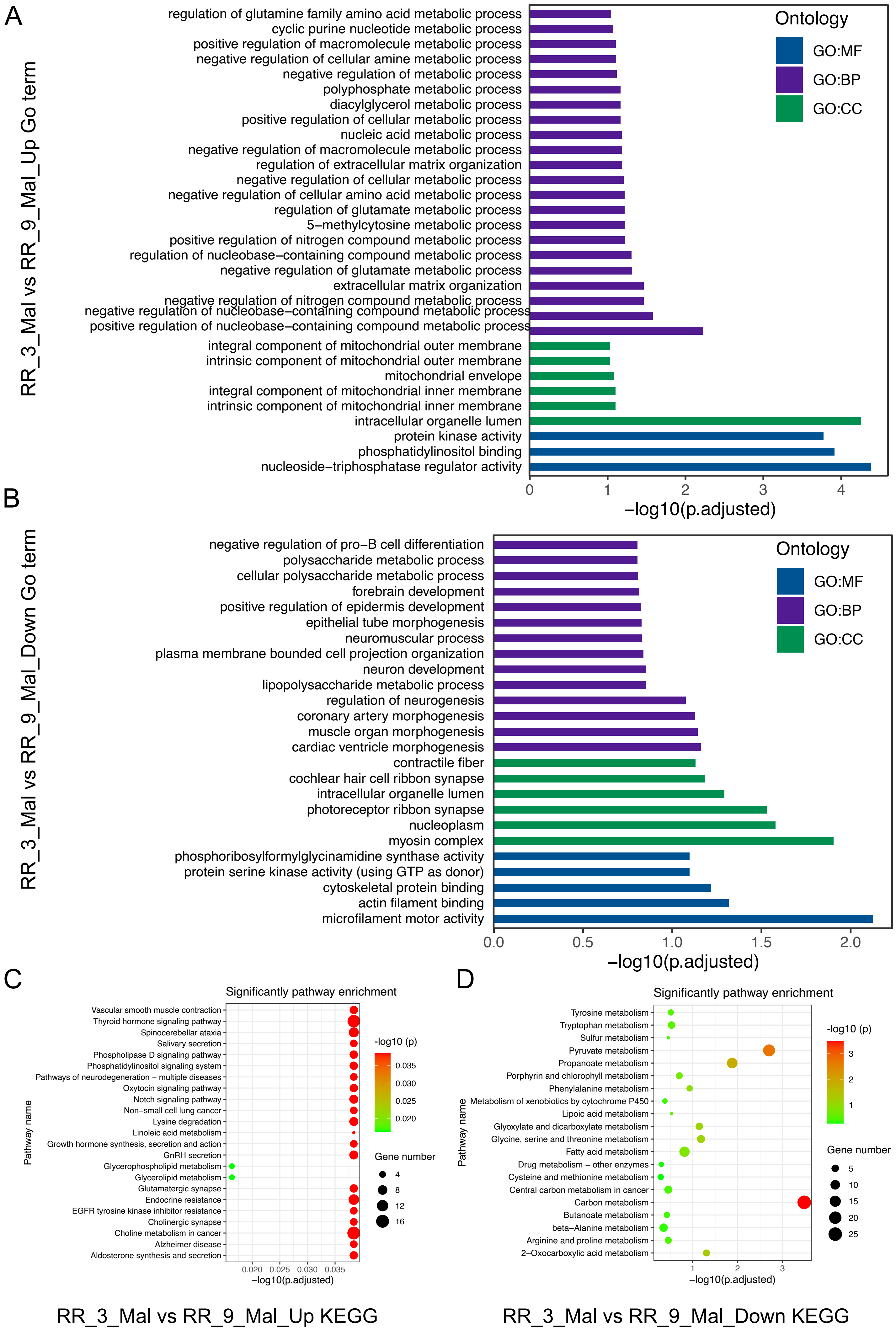
A
RR_3_Mal vs RR_9_Mal_Up Go term
regulation of glutamine family amino acid metabolic process
cyclic purine nucleotide metabolic process
positive regulation of macromolecule metabolic process
negative regulation of cellular amine metabolic process
negative regulation of metabolic process
polyphosphate metabolic process
diacylglycerol metabolic process
positive regulation of cellular metabolic process
nucleic acid metabolic process
negative regulation of macromolecule metabolic process
regulation of extracellular matrix organization
negative regulation of cellular metabolic process
negative regulation of cellular amino acid metabolic process
regulation of glutamate metabolic process
5−methylcytosine metabolic process
positive regulation of nitrogen compound metabolic process
regulation of nucleobase−containing compound metabolic process
negative regulation of glutamate metabolic process
extracellular matrix organization
negative regulation of nitrogen compound metabolic process
negative regulation of nucleobase−containing compound metabolic process
positive regulation of nucleobase−containing compound metabolic process
integral component of mitochondrial outer membrane
intrinsic component of mitochondrial outer membrane
mitochondrial envelope
integral component of mitochondrial inner membrane
intrinsic component of mitochondrial inner membrane
intracellular organelle lumen
protein kinase activity
phosphatidylinositol binding
nucleoside−triphosphatase regulator activity
0
1
2
3
4
−log10(p.adjusted)
Ontology
GO:MF
GO:BP
GO:CC
B
RR_3_Mal vs RR_9_Mal_Down Go term
negative regulation of pro−B cell differentiation
polysaccharide metabolic process
cellular polysaccharide metabolic process
forebrain development
positive regulation of epidermis development
epithelial tube morphogenesis
neuromuscular process
plasma membrane bounded cell projection organization
neuron development
lipopolysaccharide metabolic process
regulation of neurogenesis
coronary artery morphogenesis
muscle organ morphogenesis
cardiac ventricle morphogenesis
contractile fiber
cochlear hair cell ribbon synapse
intracellular organelle lumen
photoreceptor ribbon synapse
nucleoplasm
myosin complex
phosphoribosylformylglycinamidine synthase activity
protein serine kinase activity (using GTP as donor)
cytoskeletal protein binding
actin filament binding
microfilament motor activity
0.0
0.5
1.0
1.5
2.0
−log10(p.adjusted)
Ontology
GO:MF
GO:BP
GO:CC
C
Significantly pathway enrichment
Pathway name
Vascular smooth muscle contraction
Thyroid hormone signaling pathway
Spinocerebellar ataxia
Salivary secretion
Phospholipase D signaling pathway
Phosphatidylinositol signaling system
Pathways of neurodegeneration − multiple diseases
Oxytocin signaling pathway
Notch signaling pathway
Non−small cell lung cancer
Lysine degradation
Linoleic acid metabolism
Growth hormone synthesis, secretion and action
GnRH secretion
Glycerophospholipid metabolism
Glycerolipid metabolism
Glutamatergic synapse
Endocrine resistance
EGFR tyrosine kinase inhibitor resistance
Cholinergic synapse
Choline metabolism in cancer
Alzheimer disease
Aldosterone synthesis and secretion
0.020
0.025
0.030
0.035
−log10(p.adjusted)
-log10 (p)
0.035
0.030
0.025
0.020
Gene number
4
8
12
16
RR_3_Mal vs RR_9_Mal_Up KEGG
D
Significantly pathway enrichment
Pathway name
Tyrosine metabolism
Tryptophan metabolism
Sulfur metabolism
Pyruvate metabolism
Propanoate metabolism
Porphyrin and chlorophyll metabolism
Phenylalanine metabolism
Metabolism of xenobiotics by cytochrome P450
Lipoic acid metabolism
Glyoxylate and dicarboxylate metabolism
Glycine, serine and threonine metabolism
Fatty acid metabolism
Drug metabolism − other enzymes
Cysteine and methionine metabolism
Central carbon metabolism in cancer
Carbon metabolism
Butanoate metabolism
beta−Alanine metabolism
Arginine and proline metabolism
2−Oxocarboxylic acid metabolism
1
2
3
−log10(p.adjusted)
-log10 (p)
3
2
1
Gene number
5
10
15
20
25
RR_3_Mal vs RR_9_Mal_Down KEGG
