# Supplementary material for: Intrauterine Growth Restriction Induces Adulthood Chronic Metabolic Disorder in Cardiac and Skeletal Muscles
Source: Front Nutr. 2022 Jul 22;9:929943. doi: 10.3389/fnut.2022.929943 (PMC9354130; doi:10.3389/fnut.2022.929943)
Supplement: Supplementary Figure 1 — The growth curve of different treated groups. [file Data_Sheet_1.zip › supplementary material/Supplementary Table 1.docx]

**Supplementary Table 1**

| **RR_3_mal** | The rats that were given nutritional restrictions (isocaloric low protein, LP, 8%) both in utero and after birth were malnutrition at 3 months of age. We have collected 3 rats from this group. |
| --- | --- |
| **RC_3_mal** | The rats that were given nutrition restriction (isocaloric low protein, LP, 8%) in utero and were given a normal diet (*ad libitum* standard laboratory chow diet, 20% protein) after birth were malnutrition at 3 months of age. We have collected 4 rats from this group. |
| **RC_3_normal** | The rats that were given nutrition restriction (isocaloric low protein, LP, 8%) in utero and were given a normal diet (*ad libitum* standard laboratory chow diet, 20% protein) after birth were normal at 3 months of age. We have collected 4 rats from this group. |
| **CC_3_normal** | The rats that were given a normal diet (*ad libitum* standard laboratory chow diet, 20% protein) both in utero and after birth were normal at 3 months of age. We have collected 2 rats from this group. |
| **RR_9_mal** | The rats that were given nutritional restriction (isocaloric low protein) both in utero and after birth were malnutrition at 9 months of age. We have collected 3 rats from this group. |
| **RC_9_normal** | The rats that were given nutrition restriction (isocaloric low protein) in utero and were given a normal diet (*ad libitum* standard laboratory chow diet, 20% protein) after birth were normal at 9 months of age. We have collected 4 rats from this group. |
| **CC_9_normal** | The rats that were given a normal diet (*ad libitum* standard laboratory chow diet, 20% protein) both in utero and after birth were normal at 9 months of age. We have collected 3 rats from this group. |
| **RC_9_ovob** | The rats that were given nutrition restriction (isocaloric low protein) in utero and were given a normal diet (*ad libitum* standard laboratory chow diet, 20% protein) after birth were overweight at 9 months of age. We have collected 4 rats from this group. |
| **CC_9_ovob** | The rats that were given a normal diet (*ad libitum* standard laboratory chow diet, 20% protein) both in utero and after birth were overweight at 9 months of age. We have collected 3 rats from this group. |
