# Supplementary figures and images for: Intrauterine Growth Restriction Induces Adulthood Chronic Metabolic Disorder in Cardiac and Skeletal Muscles
Source: Front Nutr. 2022 Jul 22;9:929943. doi: 10.3389/fnut.2022.929943 (PMC9354130; doi:10.3389/fnut.2022.929943)

A

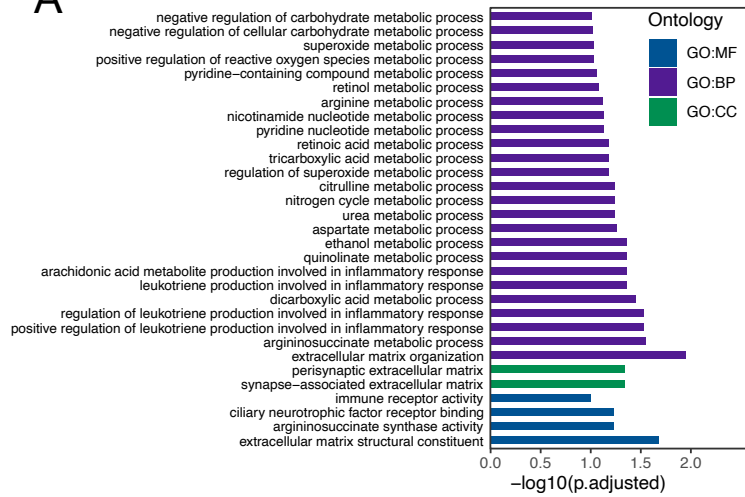

B

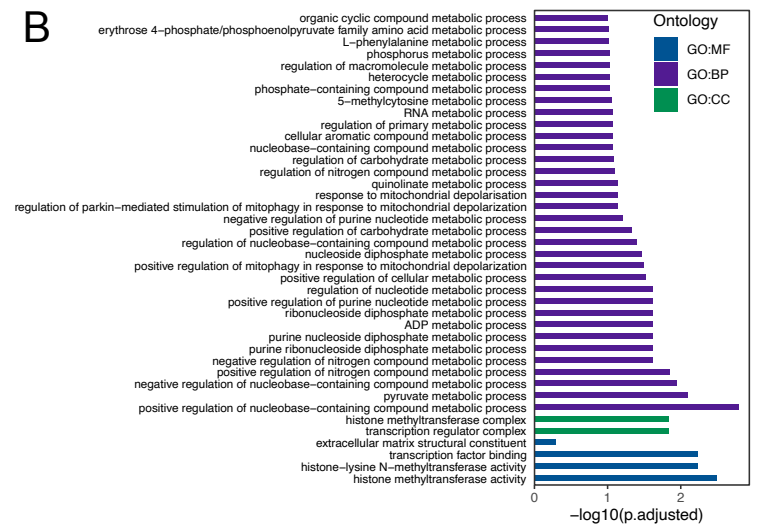

C

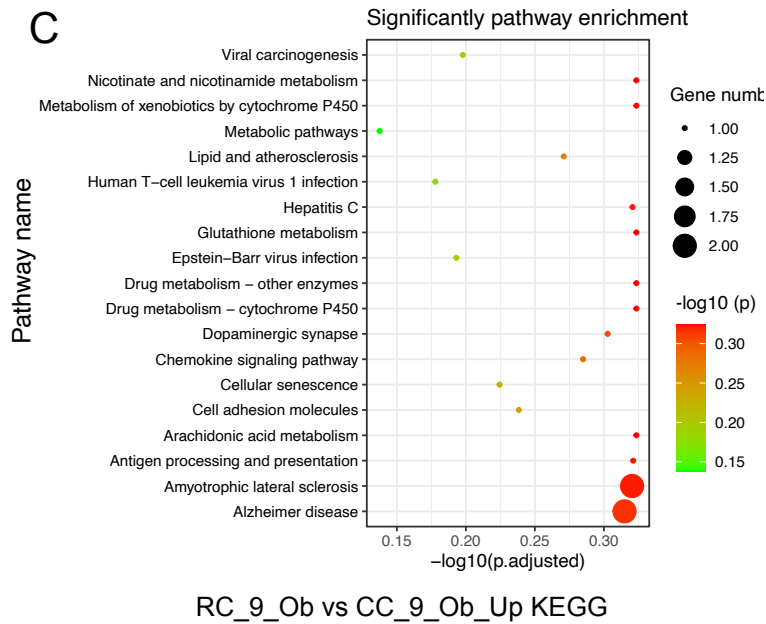

D

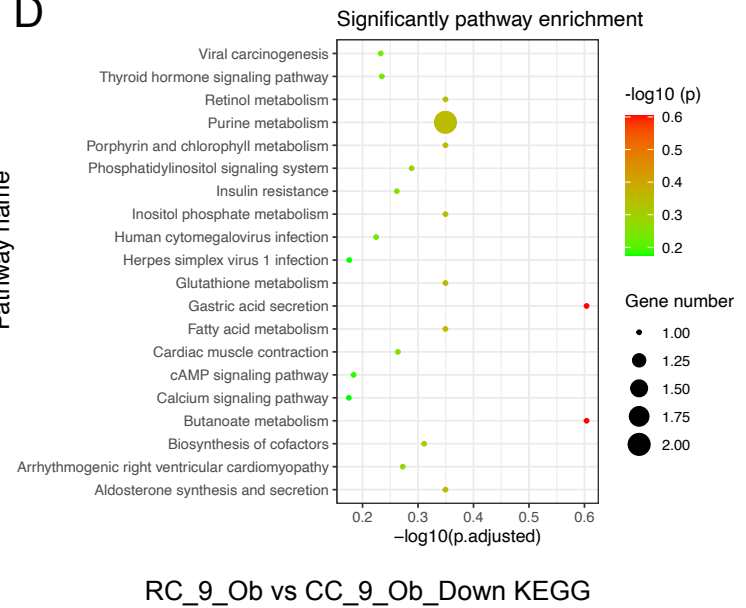

Supplement: Supplementary Figure 1 — The growth curve of different treated groups. [file Data_Sheet_1.zip › supplementary material/S_Fig_4.pdf]

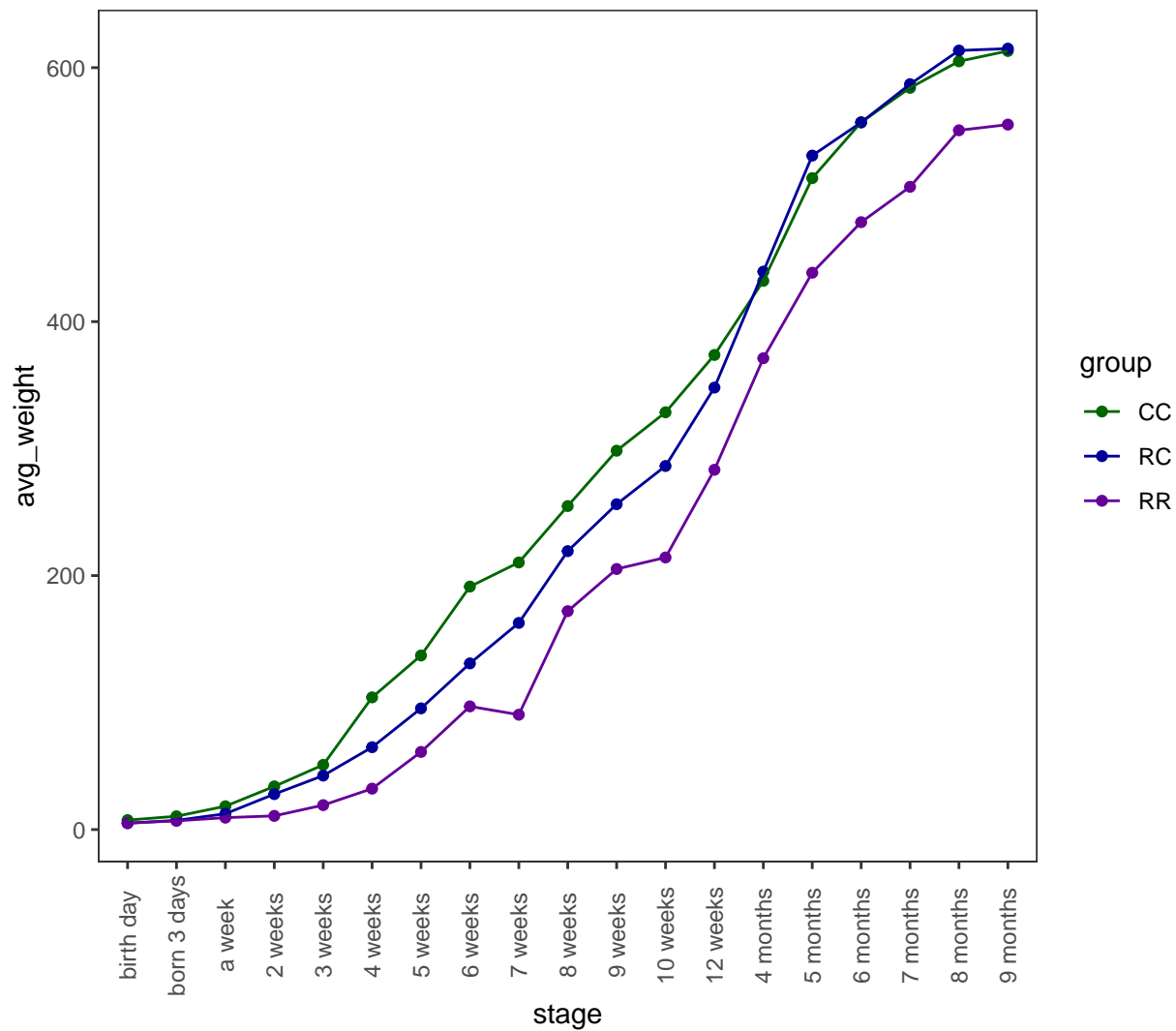

Supplement: Supplementary Figure 1 — The growth curve of different treated groups. [file Data_Sheet_1.zip › supplementary material/S_Fig_1.pdf]

A

RR\_3\_Mal vs RC\_3\_Mal\_Up Go term

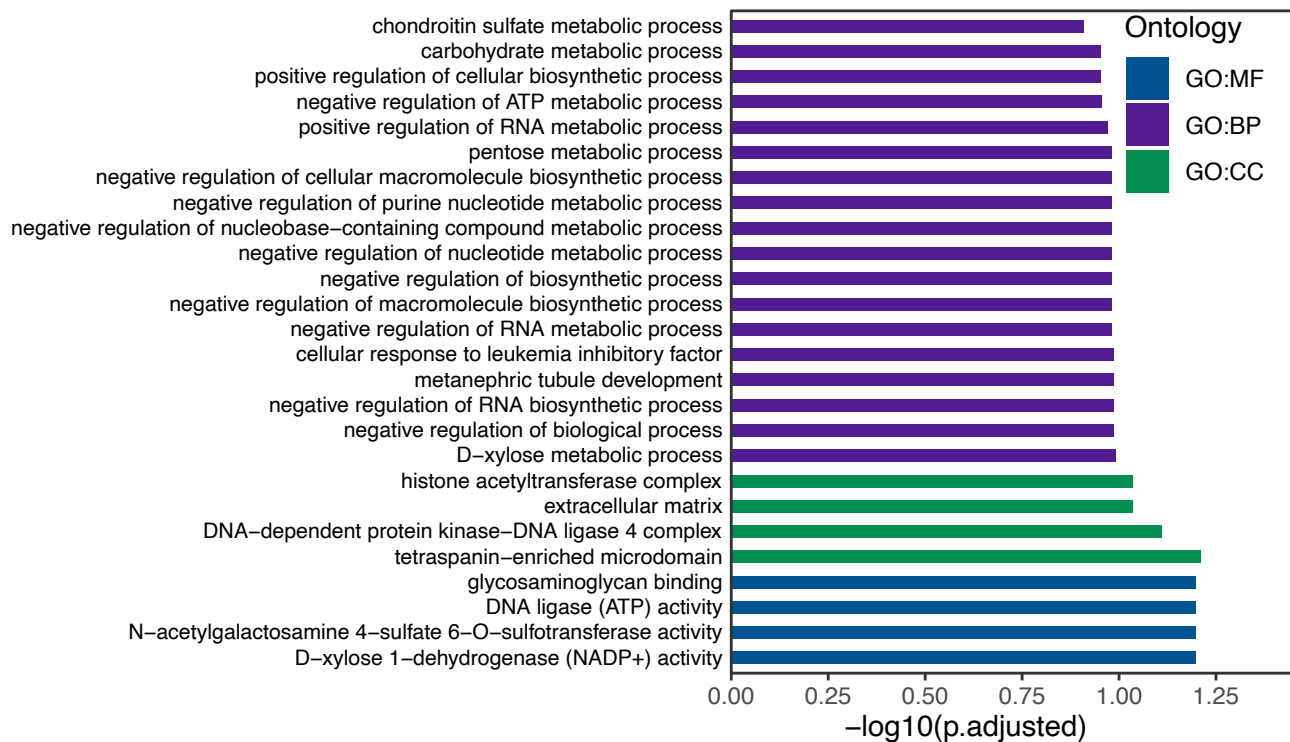

B

RR\_3\_Mal vs RC\_3\_Mal\_Down Go term

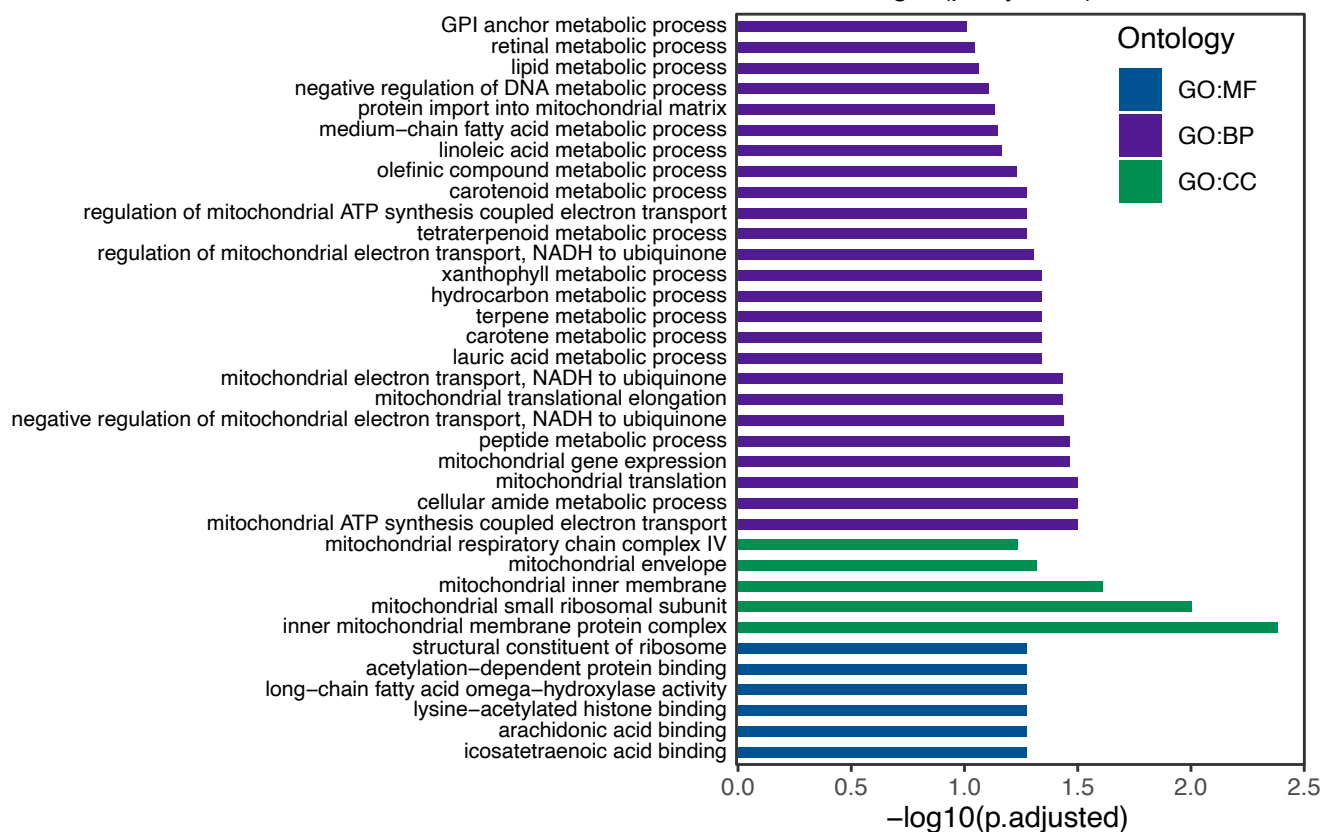

Supplement: Supplementary Figure 1 — The growth curve of different treated groups. [file Data_Sheet_1.zip › supplementary material/S_Fig_2.pdf]
