# Supplementary material for: Intrauterine Growth Restriction Induces Adulthood Chronic Metabolic Disorder in Cardiac and Skeletal Muscles
Source: Front Nutr. 2022 Jul 22;9:929943. doi: 10.3389/fnut.2022.929943 (PMC9354130; doi:10.3389/fnut.2022.929943)
Supplement: Supplementary Figure 1 — The growth curve of different treated groups. [file S_Fig_6.pdf › supplementary material/S_Fig_6.pdf]

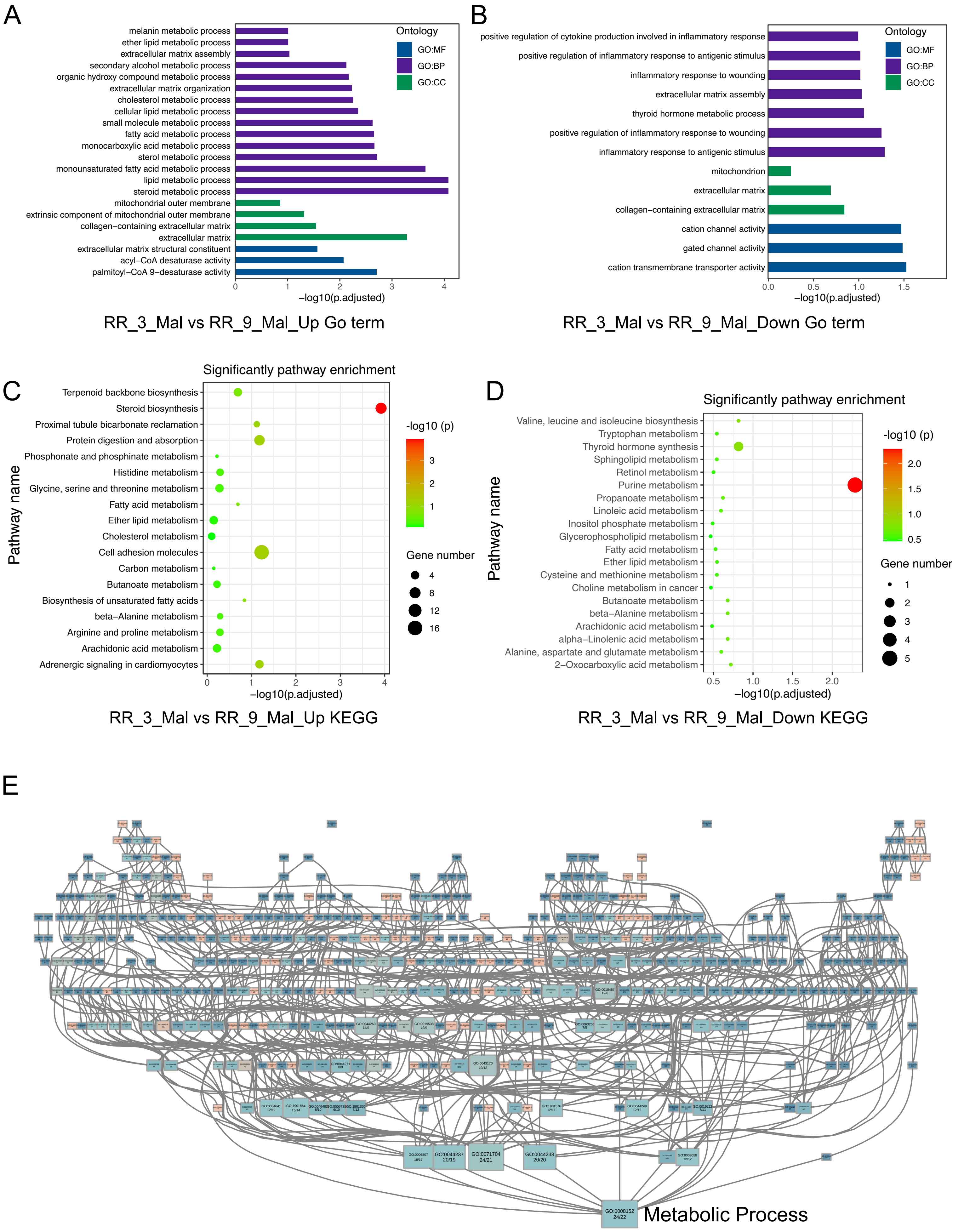
A
melanin metabolic process
ether lipid metabolic process
extracellular matrix assembly
secondary alcohol metabolic process
organic hydroxy compound metabolic process
extracellular matrix organization
cholesterol metabolic process
cellular lipid metabolic process
small molecule metabolic process
fatty acid metabolic process
monocarboxylic acid metabolic process
sterol metabolic process
monounsaturated fatty acid metabolic process
lipid metabolic process
steroid metabolic process
mitochondrial outer membrane
extrinsic component of mitochondrial outer membrane
collagen−containing extracellular matrix
extracellular matrix
extracellular matrix structural constituent
acyl−CoA desaturase activity
palmitoyl−CoA 9−desaturase activity
Ontology
GO:MF
GO:BP
GO:CC
0
1
2
3
4
−log10(p.adjusted)
RR_3_Mal vs RR_9_Mal_Up Go term
B
positive regulation of cytokine production involved in inflammatory response
positive regulation of inflammatory response to antigenic stimulus
inflammatory response to wounding
extracellular matrix assembly
thyroid hormone metabolic process
positive regulation of inflammatory response to wounding
inflammatory response to antigenic stimulus
mitochondrion
extracellular matrix
collagen−containing extracellular matrix
cation channel activity
gated channel activity
cation transmembrane transporter activity
Ontology
GO:MF
GO:BP
GO:CC
0.0
0.5
1.0
1.5
−log10(p.adjusted)
RR_3_Mal vs RR_9_Mal_Down Go term
C
Significantly pathway enrichment
Pathway name
Terpenoid backbone biosynthesis
Steroid biosynthesis
Proximal tubule bicarbonate reclamation
Protein digestion and absorption
Phosphonate and phosphinate metabolism
Histidine metabolism
Glycine, serine and threonine metabolism
Fatty acid metabolism
Ether lipid metabolism
Cholesterol metabolism
Cell adhesion molecules
Carbon metabolism
Butanoate metabolism
Biosynthesis of unsaturated fatty acids
beta−Alanine metabolism
Arginine and proline metabolism
Arachidonic acid metabolism
Adrenergic signaling in cardiomyocytes
0
1
2
3
4
−log10(p.adjusted)
-log10 (p)
3
2
1
Gene number
4
8
12
16
RR_3_Mal vs RR_9_Mal_Up KEGG
D
Significantly pathway enrichment
Pathway name
Valine, leucine and isoleucine biosynthesis
Tryptophan metabolism
Thyroid hormone synthesis
Sphingolipid metabolism
Retinol metabolism
Purine metabolism
Propanoate metabolism
Linoleic acid metabolism
Inositol phosphate metabolism
Glycerophospholipid metabolism
Fatty acid metabolism
Ether lipid metabolism
Cysteine and methionine metabolism
Choline metabolism in cancer
Butanoate metabolism
beta−Alanine metabolism
Arachidonic acid metabolism
alpha−Linolenic acid metabolism
Alanine, aspartate and glutamate metabolism
2−Oxocarboxylic acid metabolism
0.5
1.0
1.5
2.0
−log10(p.adjusted)
-log10 (p)
2.0
1.5
1.0
0.5
Gene number
1
2
3
4
5
RR_3_Mal vs RR_9_Mal_Down KEGG
E
GO:0044237
20/19
GO:0071704
24/21
GO:0044238
20/20
GO:0008152
24/22
Metabolic Process
